# The Intrinsic Cardiac Nervous System and Its Role in Cardiac Pacemaking and Conduction

**DOI:** 10.3390/jcdd7040054

**Published:** 2020-11-24

**Authors:** Laura Fedele, Thomas Brand

**Affiliations:** Developmental Dynamics, National Heart and Lung Institute (NHLI), Imperial College, London W12 0NN, UK

**Keywords:** nervous system, development, neural crest cells, innervation, ganglia, sympathetic neurons, parasympathetic neurons, neurotransmitter, neurocardiac junction

## Abstract

The cardiac autonomic nervous system (CANS) plays a key role for the regulation of cardiac activity with its dysregulation being involved in various heart diseases, such as cardiac arrhythmias. The CANS comprises the extrinsic and intrinsic innervation of the heart. The intrinsic cardiac nervous system (ICNS) includes the network of the intracardiac ganglia and interconnecting neurons. The cardiac ganglia contribute to the tight modulation of cardiac electrophysiology, working as a local hub integrating the inputs of the extrinsic innervation and the ICNS. A better understanding of the role of the ICNS for the modulation of the cardiac conduction system will be crucial for targeted therapies of various arrhythmias. We describe the embryonic development, anatomy, and physiology of the ICNS. By correlating the topography of the intracardiac neurons with what is known regarding their biophysical and neurochemical properties, we outline their physiological role in the control of pacemaker activity of the sinoatrial and atrioventricular nodes. We conclude by highlighting cardiac disorders with a putative involvement of the ICNS and outline open questions that need to be addressed in order to better understand the physiology and pathophysiology of the ICNS.

## 1. Introduction

The intrinsic cardiac nervous system (ICNS) is sometimes referred as the “little brain” of the heart [1]. It makes continuous adjustments of the cardiac mechanical and electrical activity and it consists of a network of neurons that communicate with each other and with neurons located in the extracardiac thoracic ganglia, all under the control of the central nervous system. The ICNS comprises afferent (sensory), interconnecting (local circuit), and cardio-motor (efferent sympathetic and parasympathetic) neurons [1,2]. The intrinsic cardiac neurons are mainly concentrated in intracardiac ganglia residing in specific regions of the heart, mostly in the atria, and each ganglion has a preferential region of action [2]. The activation of efferent neurons results in the modulation of the heart rate, atrio-ventricular node conduction as well as inotropism of atria and ventricles. Local circuit neurons (LCNs) work as inter- and intra-ganglionic connections, whilst afferent neurons transduce information of the cardiovascular milieu [1,3].

**Figure 1:**
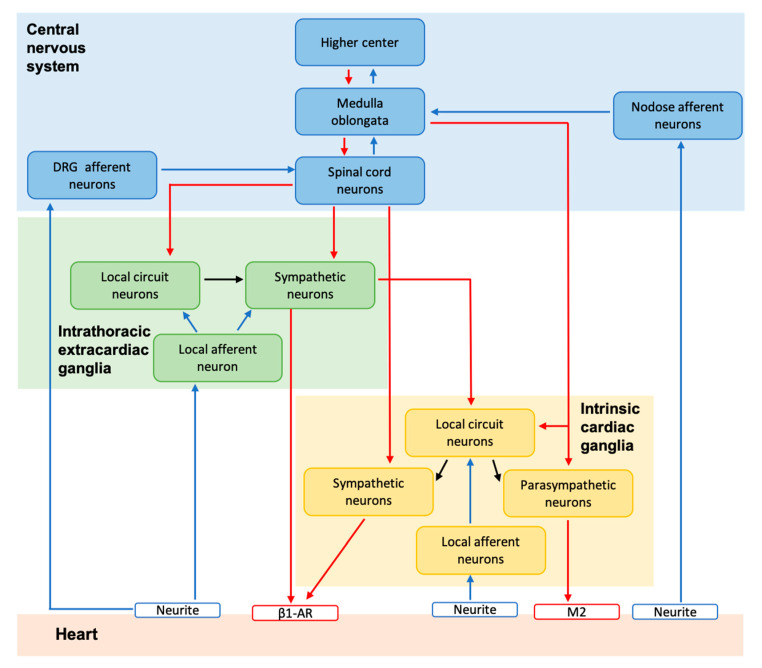
Model of the hierarchical control of the heart by the cardiac autonomic nervous system (CANS). The intrinsic cardiac nervous system (ICNS) contains efferent (parasympathetic and sympathetic) neurons, local afferent neurons and local circuit neurons. The intrathoracic extracardiac ganglia (stellate ganglia and middle cervical ganglia) contain sympathetic neurons, local afferent and local circuit neurons. The intrathoracic ganglia (intra- and extracardiac) work in close coordination as a nested loop, which is further tuned by the CNS (spinal cord, brainstem, hypothalamus, and forebrain) resulting in the regulation of cardiac function on a beat-to-beat basis. DRG: dorsal root ganglion; β1AR: beta 1 adrenergic receptor, M2: muscarinic acetylcholine receptor type 2. Neurite: local afferent neurites embedded in the cardiac walls. Figure adapted from Ardell & Armour *J. Phys* published by Wiley 2016 [2].

The control of the heart by the cardiac autonomic nervous system (CANS) is hierarchically organized and it can be subdivided into three levels (Figure 1). Level 1 is composed of the neurons in the medulla oblongata (brainstem) and spinal cord, which are controlled by higher cortical neurons (e.g., insular cortex, anterior cingulate cortex, medial prefrontal cortex, and amygdala) in the central nervous system [2,4,5]. Level 2 and 3 are in the periphery, level 2 comprises the intrathoracic extracardiac ganglia, and level 3 the ICNS [2]. Notably, the ICNS can modulate regional cardiac function in a beat-to-beat fashion and it can regulate heart function, even when disconnected from the higher levels [2]. Overall, the CANS plays a fundamental role in modulating contractility (inotropy), relaxation (lusitropy), beating rate (chronotropy), conduction velocity (dromotropy), and myocyte cohesion (adhesiotropy), and therefore affects both the electrophysiology and hemodynamics of the heart [6]. Importantly, clinical studies have demonstrated an implication of the cardiac nervous system in atrial and ventricular arrhythmias [7].

Various cardiothoracic reflexes depend on the sensory transduction of the local afferent neurons (in the intrathoracic intra- and extracardiac ganglia) as well as the afferent neurons in the spinal cord, dorsal root, and nodose ganglia. Their transductions result in the initiation of the reflex both in the central and peripheral nervous system [2]. The afferent neurons can transduce mechanical (mechanosensory neurons) and chemical (chemosensory neurons) stimuli, with the majority of the intrinsic cardiac afferent neurons transducing both modalities [3].

Cardiomyocytes and coronary vessels are regulated by the efferent neurons, as well as by circulating hormones. The efferent cardiac neuronal outputs depend on both pre-ganglionic and post-ganglionic neurons [2,8]. The sympathetic branch comprehends the pre-sympathetic efferent neurons in the brainstem that project to the pre-ganglionic neurons in the spinal cord (intermediolateral column), which, in turn synapse to the post-ganglionic sympathetic neurons residing in the intrathoracic ganglia (cervical and stellate ganglia) [2]. As depicted in Figure 1, some spinal cord neurons (although a small number) form synapses with sympathetic post-ganglionic neurons in the ICNS [2,8]. The parasympathetic pre-ganglionic neurons reside in the brainstem (mainly in the nucleus ambiguous), and project to post-ganglionic neurons that are located in the ICNS ganglia [2]. Both the sympathetic and parasympathetic efferent neurons in the ICNS are located in the major atrial and ventricular ganglia and regulate the electrical and mechanical activity of the heart [2,8].

The cardiac conduction system (CCS) plays an essential role in the initiation and coordination of the heart’s electrophysiology. It is composed of the sinoatrial node (SAN), the atrioventricular node (AVN) and the His–Purkinje system. The SAN residing in the inflow tract of the right atrium, acts as the primary pacemaker initiating the cardiac action potential. The AVN retards the conduction of the action potential from the atria to the ventricles and works as a “back-up” pacemaker in the case of a failure of the SAN. The His-Purkinje system is composed of a common bundle (the bundle of His) branching into the left and right networks of Purkinje fibers and propagates the action potential throughout the ventricles to permit their simultaneous electrical activation [9].

In this review, we will describe the role of the ICNS for cardiac electrophysiology with a particular focus on its role in modulating the function of the CCS. We discuss the embryonic development of the ICNS, the anatomy and physiological role, and its interaction with the CCS. We conclude the review with some examples of heart disease that might also involve a disruption of the ICNS and highlighting gaps that we identified in the literature that need to be addressed in the future.

## 2. Development of the Innervation of the Heart

The development of the innervation of the heart is a tightly regulated process, both spatially and temporally, which occurs concurrently with the development of the cardiovascular system [10,11]. The ICNS originates from the neuroectoderm, whereas the heart is a mesoderm derivative [12]. Cardiac innervation starts during the fifth week of human development (E10-E11 in mice) and it is characterized by four developmental stages: (1) the migration of neural crest cells (NCCs) to the dorsal aorta; (2) their differentiation into neurons; (3) the formation of the paravertebral sympathetic chain and parasympathetic cardiac ganglia through migration and aggregation of neuronal precursors; and (4) patterning and differentiation of the neuronal projection in the heart [13]. The parasympathetic innervation of the heart becomes functional before sympathetic neurons start to differentiate. The developing heart is initially relying on intrinsic cardiac adrenergic (ICA) cells as a source of catecholamines [10]. Cholinesterase activity appears in cardiac neurons between the fourth and seventh month of gestation in humans and at postnatal day 4 (P4) in rats; while, acetylcholine appears at E19 in rats [14,15].

### 2.1. Intrinsic Cardiac Adrenergic Cells (ICA)

Catecholamines play a key role in cardiac embryonic development, even in the pre-innervation stages and before chromaffin cells of the adrenal glands can synthesize them (E15.5 in mice). Targeted disruption in mice of the genes encoding the enzymes that are involved in catecholamine biosynthesis, dopamine β-hydroxylase (DBH), and tyrosine hydroxylase (TH) are embryonic lethal [16,17]. In the early embryo (E8.5 in mice), the myocardium itself expresses the catecholamine synthesizing enzyme phenylethanolamine-*N*-methyltransferase (PNMT), with the ICA cells being the major source of catecholamine synthesis [18].

ICA cells are transiently found during development, their fate is not clear, and some might differentiate into pacemaker cells [13]. In accordance with this, in the mouse embryo, ICA cells are present in regions that are associated with the CCS, which suggests a specific role in its development [19,20]. Interestingly, some ICA cells persist in the adult ventricular myocardium, and they might act as a “backup” system when innervation is lost [21]. In fact, after denervation due to cardiac transplantation, the number of ICA cells increased along with the expression of TH, DBH, and PNMT. ICA cells might have a cardioprotective role, as they also express cardioprotective genes, e.g., calcitonin gene related peptide (CGRP) [22,23].

### 2.2. Migration of Neural Crest Cells

Neural crest cells (NCCs) migrate into the developing heart in the fifth week in humans (E8.5 in mice). Depending on their region of origin, they give rise to distinct cell types, among them the sympathetic, sensory, and parasympathetic neurons [10].

Sympathetic neurons originate from the trunk NCCs that migrate along the ventral pathway towards the dorsal aorta; they then migrate into rostral and caudal direction to form the paravertebral sympathetic chain. On their migratory route, the NCCs are guided by attracting and repelling factors, among them ephrins and semaphorins (SEMA) [13]. Ephrins orchestrate the direction of the migration along the ventral pathway: EphrinB, present in the posterior side of the somite, has a repulsive effect on NCCs, which express the EphB2 receptor [24]. The migration towards the dorsal aorta is directed by SEMA acting on its receptors (e.g., Neuropilin-1/2), which are expressed in NCCs. SEMA3C is expressed in the myocardial cuff cells in the outflow tract and it acts as an attractive cue for NCCs, contrasting to the action of the repelling SEMA6B and SEMA6B present in the dorsal neural tube. In accordance with the key role of SEMA in the migration of NCCs destined to form sympathetic neurons, *Nrp1* and *Sema3a* knock-out mice display a sinus bradycardia phenotype as a result of sympathetic ganglia dysfunction, e.g., ectopic sympathetic ganglia and malformed stellate ganglia [25].

**Figure 2:**
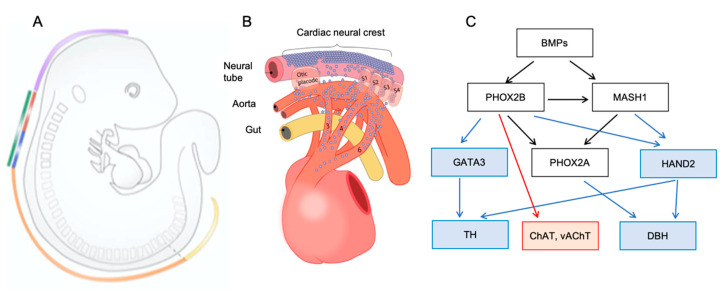
(**A**) Regionalization of the neural crest along the dorsal neural tube of the mouse embryo (E10.5): cranial (purple), vagal (green), trunk (orange), and sacral (yellow). Vagal neural crest cells (NCCs) are further subdivided into: cardiac (red) and enteric (blue) lineages. (**B**) Migration of cardiac NCCs through or lateral to the somites into the branchial arch arteries and further into the outflow tract. (**C**) Transcriptional network involved in the specification and differentiation of NCCs into sympathetic and parasympathetic neurons. White box: factors involved in both pathways; blue: factors involved in the differentiation of sympathetic neurons and red: of parasympathetic neurons. Abbreviations: BMPs- bone morphogenic proteins; ChAT- choline acetyltransferase; DBH- dopamine β-hydroxylase; GATA3- GATA binding protein 3; HAND2: heart and neural crest derivative expressed 2; MASH1- mammalian achaete-scute homologue; PHOX2 (A or B)- paired-like homeobox 2A or 2B; TH- tyrosine hydroxylase; vAChT- vesicular acetylcholine transporter. (**A**) reproduced with permission from Hutchins, E.J., et al. *Dev. Biol* published by Elsevier 2018 [29]; (**B**) reproduced with permission from Végh A.M.D., et al. *JCDD* by MDPI 2016 [10]; (**C**) adapted from Végh A.M.D., et al. *JCDD* by MDPI 2016 [10].

The cardiac parasympathetic ganglia originate from the so-called “cardiac NCCs”, which is a subset of the vagal neural crest (Figure 2). In contrast to the sympathetic counterparts, they migrate either through or lateral to the somites, entering the heart and forming the cardiac ganglia from E12.5 onwards [26]. Interestingly, NCCs are not the only source of parasympathetic neurons, as they can also originate from the nodose placode. This was demonstrated by the ablation of the NCCs in quail to chick chimeras and by Wnt1-Cre lineage tracing in mice [26,27]. Further research is needed in order to elucidate the potential role of guidance cues in the migration of the parasympathetic neurons, as some factors have been proposed, purely based on their transient immunoreactivity during development, e.g., HNK1 (human natural killer-1/CD57) in regions with ingrowing parasympathetic fibers in the basal region of the heart [28].

### 2.3. Differentiation of Neural Crest Cells

After NCCs have reached the dorsal aorta (E9.5) and the outflow tract (E10.5), they encounter various factors that are involved in their specification towards sympathetic or parasympathetic neuronal identity [10]. It has been suggested that sympathetic and parasympathetic neurons share a common precursor, with a potential role of bone morphogenetic protein (BMP)2 in their specification. In accordance with this, sympathetic neurons can transdifferentiate in vitro into parasympathetic neurons upon stimulation with low BMP2 levels [30]. High levels of BMP2 favors sympathetic differentiation, whilst low levels induce parasympathetic identity [10].

The initial steps in the differentiation process are similar between sympathetic and parasympathetic neurons (Figure 2). BMPs that are released by the epicardial and vascular cells elicit a transcriptional differentiation cascade, which is shared by both, sympathetic and parasympathetic neuronal precursors. The disruption of BMPs results in compromised differentiation into sympathetic neurons. For example, inhibition of BMP4-7 in avian embryos prevents sympathetic differentiation and sympathetic neuronal progenitors die after they have reached the dorsal aorta in *Alk3* (the type 1 BMP receptor) conditional knockout mice [31,32]. Regarding parasympathetic neurons, BMP5 and 7 are present in ciliary neurons [33]. In both sympathetic and parasympathetic neuronal differentiation, BMPs initiate the expression of paired-like homeobox 2b (PHOX2B) and the mammalian *achaete-scute* homolog (MASH1). Accordingly, *Phox2b* and *Mash1* knockout mice display a disruption of the development of all autonomic ganglia [34,35,36]. PHOX2B and MASH1 have a key role in the induction of the expression of the enzymes that are involved in catecholamine biosynthesis, TH, and DBH (Figure 2). The former through the action of PHOX2B via GATA3, whilst the latter through the action of PHOX2B and MASH1 via PHOX2A and heart and neural crest derivative expressed 2 (HAND2) [10]. *Gata3*^−/−^ and *Hand2*^−/−^ mice die in utero (E9.5-E11) and they both display a reduction of *Th* and *Dbh* expression [37,38,39]. Likewise, mice with a specific deletion of *Hand2* in NCCs also displayed an impaired formation of sympathetic neurons [37,38,39].

Studies using ciliary ganglia have reported that parasympathetic neurons do not express GATA3 and HAND2, nor do they synthesize catecholamine enzymes [33]. By contrast, they express the parasympathetic markers choline acetyltransferase (ChAT) and vesicular acetylcholine transporter (vAChT), possibly via a pathway downstream of PHOX2B through the action of the glial cell line-derived neurotrophic factor GDNF/Ret, as shown in cranial parasympathetic ganglia [10,40]. In accordance with a role of the signaling pathway downstream of GDNF and the involvement of the GDNF receptor Ret, E18 *Ret* knockout mice present a reduction in the volume of cardiac ganglia together with decreased innervation in the ventricular CCS [41].

The differentiation of cardiac parasympathetic neurons has not been extensively studied. The extrapolation or conclusions drawn from research done on ciliary and/or cranial parasympathetic ganglia might not be very accurate and, therefore, more research is needed in this regard.

### 2.4. Survival and Patterning

Once the target destinations have been reached, both sympathetic and parasympathetic neurons require various factors that are released by cardiomyocytes, vascular smooth muscle cells, and glial cells in order to ensure that the neurons are matched to their targets and to prevent apoptosis [10].

In the case of sympathetic neurons, nerve growth factor (NGF) and neurotrophin-3 (NT-3) are among the most important trophic factors [10]. NGF is secreted in its proform (proNGF) and extracellularly cleaved by neuron-specific matrix metalloproteinases, with the ratio of proNGF to NGF determining the cell fate [42,43]. ProNGF in fact, has a high affinity to the neurotrophin receptor p75 (NGFR), which triggers the apoptotic pathway with minimal activation of the survival pathway mediated by NTRK1 (TRKA). In contrast, NGF has a higher affinity to NTRK1, hence triggering the “survival” signaling [42]. The transient expression (E13.5–E15.5) of NGF from venous smooth muscle cells dictates the pattern of sympathetic growth. It directs the nerve growth along the subepicardial layer of the developing heart, establishing an epicardial-endocardial gradient [10]. NGF is not only produced by vascular smooth muscle cells, but is also synthesized by cardiomyocytes. The expression of NGF is upregulated by endothelin-1 (ET-1), which plays a key role in sympathetic innervation. Accordingly, *Et1*^−/−^ mice display a significant reduction of NGF expression and a decrease in the number of stellate ganglia. The defects can be rescued by the forced expression of cardiac NGF [44]. The NGF levels decrease at birth (P0), but reach a second peak at P8 and a low level persists from P21 onwards (in rats) [10]. In addition to neuronal survival/patterning, NGF is also involved in the formation and strengthening of the neurocardiac varicosities between sympathetic neurons and cardiomyocytes [45,46]. These varicosities and neurocardiac junction will be addressed in more details in Section 5.

NT-3 is secreted from smooth muscle cells of blood vessels that are located adjacent to sympathetic ganglia. During sympathetic neuronal development in rats, there is a switch from the NT-3 to the NGF pathway. This is consistent with the developmental regulation of their respective receptors: NTRK3 (TRKC), which is expressed in early stages, whilst NTRK1 (which binds to NGF) from E15 [47]. *Nt3*^−/−^ and *Ntrk3*^−/−^ mice show similar cardiac defects, but *Nt3*^−/−^ mice present a more severe loss of sympathetic ganglia, indicating that other receptors might also be involved [48]. Interestingly, the sympathetic neuronal loss occurs at P0, and there are no differences in neuroblast numbers at E15 between the knockout and wild-type counterparts [49]. These results would suggest either a concomitant role of the two trophic factors; potential differences in developmental expression between rats and mice or other compensatory mechanisms should not be excluded.

The four glial cell-line-derived neurotrophic factor family of ligands (GFLs) and their receptors are involved in parasympathetic neuron survival. Despite their name, they are also expressed in cardiomyocytes [50]. GFLs are produced as preproGFL, followed by a cleavage of the signal sequence to generate proGFL. The activation of proGFL possibly requires another cleavage, but the specific proteases that are involved are yet to be identified [51]. The four ligands are called glial cell line derived neurotrophic factor, neurturin, artemin, and persephin and they act on their respective receptors: GFRa1, GFRa2, GFRa3, and GFRa4 [51]. The GFRas work as co-receptors of the receptor tyrosine kinase RET [51]. Ligand binding elicits the activation of downstream signaling, e.g., the inhibition of apoptosis and promotion of cell survival via the AKT signaling pathway [52]. Ret and GFRa2 are highly expressed in cardiac ganglia at E18 and P21 in rat hearts. The role of these signaling pathways in cardiac innervation is evident by studies using knock-out mice: neurturin knock-out (*Ntn*^−/−^) mice exhibit cardiac innervation defects and *Gfra2*^−/−^ mice display reduced innervation in both ventricles (by 40%) and in the ventricular CCS (by 60%) [41,53,54].

Apart from the GDNF family, there is some evidence of the potential involvement of NGF in the survival of parasympathetic neurons. The NGF receptor NTRK1 was, in fact, identified in adult cholinergic cardiac ganglia, and both sympathetic and parasympathetic ganglia are lost in *Ngf*^−/−^ mice [55,56,57]. From a more functional perspective, it has been shown that in vivo application of NGF in mice potentiates the excitability of the parasympathetic neurons, it also decreases their cumulative afterhyperpolarization in vitro and increases their dendritic growth in vivo [58]. The effects of NGF on neurite growth were also shown while using dissociated parasympathetic ciliary ganglia [59].

## 3. Anatomy of the ICNS

Cardiac innervation was first studied in the myocardium of frogs and various large mammals (dogs, lambs, cats, monkey) while using bright field microscopy [60,61]. Methylene blue staining permitted the visualization of neurons, their ramification, and the identification of ganglionated plexi (GP) in the heart in various species, including humans [62]. GP are defined as a cluster of neuronal somata and nerve fibers [7]. The wider employment of electron microscopy allowed for the ultrastructural investigation of the neuronal organization of both atrial and ventricular walls [62,63,64] as well as providing the first evidence for a neuro-cardiac communication [65,66,67]. In the last 50 years, immunohistochemistry/immunofluorescence studies allowed for the characterization of the innervation of the heart during development, their higher density in specific regions of the heart (e.g., CCS), the transmural pattern of innervation [68], and differences in health and disease [64,69,70].

**Figure 3:**
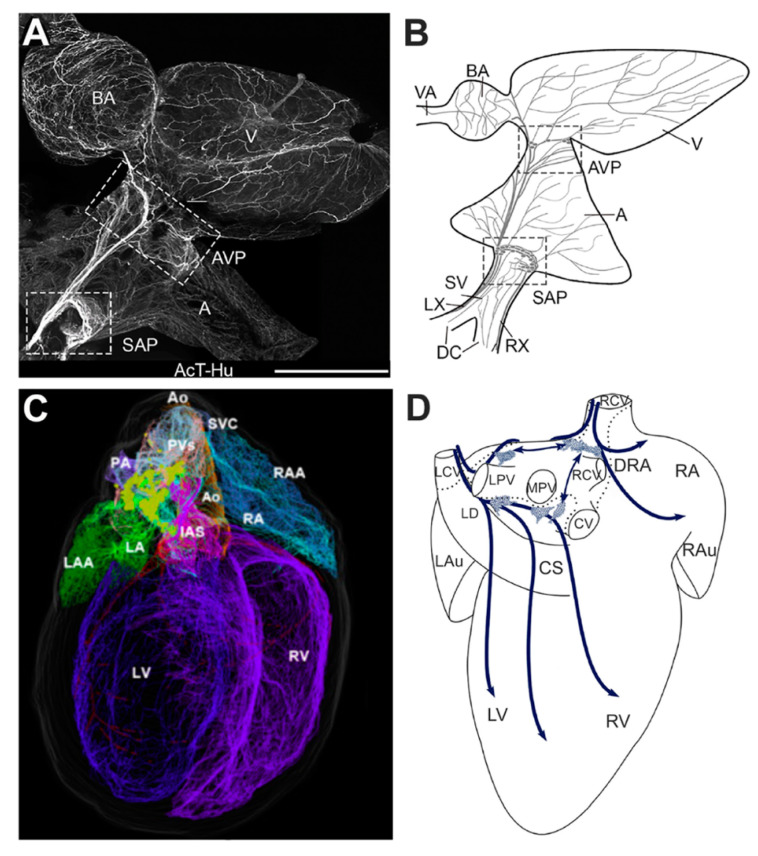
Intracardiac innervation of the zebrafish, rat and mouse hearts. (**A**) Whole mount immunostaining using antibodies directed against acetylated tubulin (AcT) and human neuronal protein (Hu) to label the ICNS of the zebrafish heart. (**B**) Schematic overview of the major elements of cardiac innervation. Boxed areas: sinoatrial plexus (SAP) and atrioventricular plexus (AVP). Abbreviations for **A** and **B**: A—atrium; AVP—atrioventricular plexus; BA—bulbus arteriosus; DC—ducts of Cuvier; LX and RX, left and right vagosympathetic trunks; SAP- sinoatrial plexus; SV—sinus venosus; V—ventricle; VA—ventral aorta (to the gills). (**C**) Posterior view of a 3D reconstructed male rat heart showing the context, extent, and distribution of the intrinsic cardiac neurons (yellow labelled), located on superior and posterior surfaces of the atria (**D**) Schematic overview of the intracardiac ganglia in the mouse heart, dorsal view. Dotted lines delineate the heart hilum, polygonal areas the main locations of the cardiac ganglia. Abbreviations for **C** and **D**: Ao- aorta; CS- coronary sinus veins; CV—caudal vein; DRA—dorsal right atrial; IAS—interatrial septum; LA—left atrium; LAA (or LAu)—left auricle; LCV—left cranial; LD—left dorsal; LPV—left pulmonary; LV—left ventricle; MPV—middle pulmonary veins: PA—pulmonary artery; PVs—pulmonary veins; RA—right atrium; RAA (or Rau) —right auricle; RCV—right cranial (superior caval) vein; RV—right ventricle; SVC—superior vena cava. Panel (**A**-**B**) reproduced with permission from Stoyek, M.R. et al., *J. Comp. Neurol.* Published by Wiley, 2015 [78]. (**C**) Reproduced with permission from Achanta, S. et al. *iScience* published by Elsevier, 2020 [81] (**D**) reproduced with permission from Rysevaite, K. et al., *Heart Rhythm* published by Elsevier, 2011 [74].

These anatomical studies have elucidated the location of GP, the majority of them are located in supraventricular regions, either on the epicardium or embedded in fat pads on the surface of the heart hilum [7]. The distribution of GPs is different across species, they are more scattered in larger (e.g., sheeps, pigs) [71,72] when compared to smaller mammals (e.g., mice and rats), with a lower density of innervation of the cell bodies in smaller mammals [7,73,74] (Figure 3). Moreover, intracardiac ganglia in the ventricles are only present in larger mammals, with innervation originating from atrial ganglia [7,73]. In larger mammals (e.g., rabbit, dog, sheep, and humans, the GP can be located in 5–7 subregions: dorsal right atrial, ventral right atrial, ventral left atrial, left dorsal, middle dorsal, right coronary, left coronary [7] (Figure 3). For a more detailed comparison across species of the intrinsic innervation of the heart, refer to Wake and Brack 2016 [7]. The left and right coronary GP emerge from the arterial regions, the dorsal right atrial GP from either the right caudal vein or the vena cava, the middle dorsal GP is located between the pulmonary veins with some contact with the left dorsal, and the ventral ganglia are positioned ventrally from the left pulmonary vein [7,11]. From an anatomical perspective, the SAN is innervated by post-ganglionic fibers from the ventral and dorsal right atrial, whilst the AVN from the middle dorsal and in part from the left dorsal [11].

Recent studies have established the isolated zebrafish heart as an alternative model for studying the autonomic modulation of the primary pacemaker of the zebrafish, which is a ring-like structure termed the sinoatrial ring (SAR), in order to address the limitations that are present in larger animals [75,76] (Figure 3). Using the zebrafish heart preparation with an intact intrinsic and extrinsic innervation, it has been shown that cardiac pacemaking can be modulated by both sympathetic and parasympathetic pathways. This work has also revealed that the zebrafish pacemaker cells express both muscarinic acetylcholine (M2) and β2-adrenergic receptors [75]. Similar to mammals, most of the zebrafish intracardiac ganglia are located adjacent to the SAR and they are immunoreactive to ChAT, some to TH, together with other “non-classical” neurotransmitters (e.g., nitric oxide) [77]. These ganglia are innervated by the right and left vagus nerve. Moreover, neuronal somata in the ganglia projecting back to the central nervous system were observed, possibly acting as afferent neurons providing a link between the ICNS and central nervous system [78]. The zebrafish has been widely employed in cardiovascular research [79]. It has become a powerful model, as the human and zebrafish genomes are similar (70% of the protein-coding human genes are found in zebrafish and 84% of human disease genes have a zebrafish counterpart) [80]. Despite significant anatomical differences, the functional properties of the zebrafish heart (e.g., comparable heart rate, action potential morphology) are surprisingly similar the human heart [79]. The zebrafish genome has been fully sequenced and it can be easily manipulated and, in addition, cardiac function can be examined in vivo [77].

### 3.1. Morphology and Electrophysiological Properties of Cardiac Ganglia

Intracardiac ganglia are heterogeneous and they also include the small intensely fluorescent (SIF) cells that can have various functions: endocrine, chemoreceptive and interneuronal [82]. On the basis of their three-dimensional cell shape, size, and acetylcholinesterase (AChE) staining, Pauza and co-workers distinguished them into globular and plain ganglia. The former are more densely packed, containing 100–200 neurons occupying 0.01–0.17 mm^2^, whilst the latter present a more intense staining for AChE, neurons reside side-by-side and they contain no more than 50 cells [83]. The size of neuronal somata is similar to the ones in other autonomic ganglia: 15–30 μm in the short axis and 20–45 μm in the long axes [83] and they are classified into large (80%) and small neurons (20%) [84].

**Figure 4:**
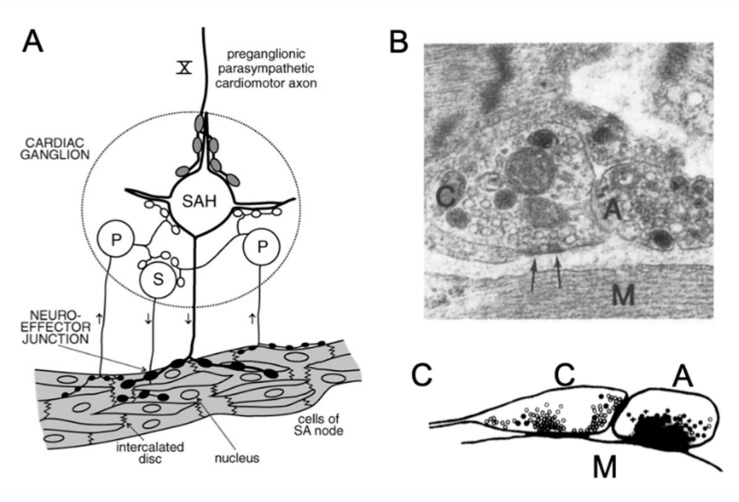
Neurocardiac transmission in the sinoatrial node (SAN). (**A**) Diagram illustrating the distinct cell types of the intracardiac ganglia and their putative roles: SAH (slow afterhyperpolarization) cells are the principal cells and the only ones receiving pre-ganglionic inputs. S (somatic) cells and some SAH cells can receive inputs from P (pacemaker) cells. P cells are putative sensory neurons. (**B**) Electron micrograph of the neurocardiac junction at the level of the sinus venosus of the toad. Cholinergic (C) and adrenergic (A) varicosities in contact with a cardiomyocyte (M). No membrane specializations are visible except for an electron dense area as indicated by the arrows. (**C**) Reconstruction of the neurocardiac junction depicted in (**B**). Cholinergic vesicles are present in the prejunctional membrane facing the cardiomyocyte and the adrenergic varicosity, respectively whilst adrenergic vesicles are mainly present in close opposition to the cardiomyocyte. The black area between the neuronal varicosity and the cardiomyocyte represents the cleft. Panel (**A**) reproduced with permission from Jänig, W., *J. Physiol.* published by Wiley, 2011 [91]. Panel (**B**,**C**) reproduced with permission from Klemm, M. et al., *J. Auton. Nerv. Syst.* published by Elsevier, 1992 [92].

According to their morphological and electrophysiological features, intracardiac neurons have been classified into type I (or phasic, also known as somatic (S) cells) and type II (or tonic). Type II neurons are further subclassified into slow afterhyperpolarization (SAH) cells, and pacemaker (P) cells) (Figure 4) [85,86,87,88]. S-cells receive local excitatory inputs; they have small somata and are monopolar; their action potentials have a phasic profile that is characterized by brief afterhyperpolarizations [85,86]. SAH and P cells are both defined by their prominent afterhyperpolarization. SAH cells are multipolar principal neurons; they receive strong efferent connections from the vagus and present a tonic profile [85]. P cells (or pacemaker cells) are neurons that present a pseudounipolar or bipolar morphology. They are named after their electrophysiological profile: they present an hyperpolarization-activated inward current that is similar to the one in cardiac pacemaker cells [85]. Interestingly, immunostaining with synaptophysin to visualize synaptic boutons revealed that P-cells were the only types that did not receive any synaptic inputs and may act as sensory neurons [86]. Using a working heart-brainstem preparation, McAllen and colleagues further reported that about 40% of intracardiac neurons received vagal inputs (principal cells) (Figure 4). The remaining were classified as quiescent, as they did not respond to vagal stimulation, supporting their role as interneurons or sensory neurons [89]. Contrasting to the above subdivision, Rimmer and Harper did not find any spontaneous rhythmic activity (i.e., in P-cells) and classified the intracardiac neurons on the basis of their excitatory post-synaptic potentials into phasic, multiply adapting, and tonic neurons [90].

### 3.2. Neurochemical Characteristics

Various immunohistochemical studies have reported the immunoreactivity of intracardiac ganglia to various neuromodulators and neurotransmitters, debunking the former belief that these were purely post-ganglionic parasympathetic efferent neurons [7]. They were found to stain positive for cholinergic (choline acetyltransferase, ChAT), adrenergic (TH), as well as putative sensory neuronal (substance P and CGRP) markers along with other co-transmitters, such as neuropeptide Y (NPY), neuronal nitric oxide synthase (nNOS), and vasoactive intestinal peptide (VIP) [7]. The functional relevance of these co-transmitters is discussed in more detail in Section 5.3. The majority of ganglia in various species (e.g., mouse, rat, guinea pig, human) were immunoreactive to ChAT [93,94,95]. It has been shown that cholinergic ganglia innervate both the sinoatrial and atrioventricular regions as well as the ventricles, supporting evidence for the parasympathetic regulation of the ventricles. TH immunoreactivity was found in both small intensely fluorescent cells as well as in larger neurons [96,97]. SIF cells are either present as small cell clusters in large ganglia or dispersed across the cardiac walls. There is some contrasting evidence on the percentage of ganglia positive to TH, with some studies reporting their absence in P neurons and presence in fibers [95], whilst others showing staining in larger neurons [98]. Notably, a study on human intrinsic cardiac ganglia reported a colocalization of TH and the vesicular monoamine transporter 2 (VMAT-2) [99]. VMAT-2 is usually required for catecholamine storage and release. This colocalization suggests the ability of a subset of the intrinsic cardiac neurons to synthesize, store, and release catecholamines [99] Interestingly, several investigators have reported a group of neurons (10–20%) that are labelled with both ChAT and TH antibodies [94,100].

Contrasting to the atria with higher levels of ChAT immunoreactivity in the somata and fibers, the ventricles present a dominance of TH labelled fibers [97]. There is some variability across various studies on the proportion of nNOS immunoreactive cells, but mostly they are co-localized with ChAT neurons [7,95,99] consistent with the role of NO as co-transmitter in parasympathetic neurons [101]. A number of studies found VIP to be expressed mainly in neuronal fibers, with immunoreactivity in neuronal somata being absent or present in a low number of neurons [99,102,103]. NPY is co-released with norepinephrine (NE) in sympathetic neurons [104] and is found to co-localize in TH, nNOS, and ChAT immunoreactive neurons [7,95]. Substance P (SP) and CGRP are generally considered to be markers of afferent neurons, i.e., neurons that monitor the chemical and physical state of the myocardium, for example, giving feedback about the heart rate. They have a similar pattern, although SP is less abundant [94]. The number of SP or CGRP immunoreactive neuronal fibers is higher near the heart hilum with various bifurcations near the atrial regions and the roots of the pulmonary veins. SP-positive fibers were found to be around nNOS and ChAT immunoreactive somata, possibly suggesting an afferent role related to cholinergic and nitrogenic neurons [7]. Notably, the ICNS contain no or very little cell bodies that are immunoreactive to SP and CGRP. These findings suggest that the cell bodies of these immunoreactive afferent fibers reside either in the nodose or stellate ganglia [7], although one study identified SP and CGRP somata in the ICNS of the guinea pig [84]. On the basis of their molecular profile and spatial distribution, Achanta and coworkers have recently classified the intracardiac neurons in various clusters employing laser captured neuronal microdissection paired with RT-qPCR. Consistent with the previously reported immunohistochemical characteristics, in some neurons some genes were expressed in a pairwise fashion, e.g., neuropeptide Y and tyrosine hydroxylase [81]. However, the biophysical characteristics are yet to be correlated with the molecular profile of each class of neurons. This could be, for example, undertaken while using the Patch-seq technology [105] either in cultured cardiac neurons or from neurons patched from the intact atrial preparation. This technology would allow for the analyses of the neuronal biophysical profile using whole-cell patch clamp, followed by the aspiration of the somatic compartment for single-cell RNA seq analysis [105].

## 4. Physiological Role of Innervation in the CCS

The neurochemical heterogeneity of the intrinsic cardiac neurons that are mentioned in Section 3.2 provide evidence for different neuronal populations, suggesting the presence of efferent cholinergic and adrenergic neurons all in close vicinity to the afferent fibers [7,106]. In this section, we will discuss their function in the modulation of the conduction system, through their direct action, their interactions within the ICNS, or with the intrathoracic extracardiac neurons.

### 4.1. Direct Action of the Cardiac Ganglia on the CCS

A functional contribution of both sympathetic and parasympathetic intracardiac efferent neurons has been demonstrated, employing the decentralized and non-decentralized canine heart [107] and the Langendorff-perfused rabbit hearts [108]. Specific stimulation of selective loci in intracardiac ganglia (with nicotine) or with electrical stimulation elicited bradycardia, tachycardia, or bradycardia, followed by tachycardia. These effects depended on the activation of the intracardiac ganglia through nicotinic agonists and they were abolished in the presence of the hexamethonium [108]. The bradycardic effects were mediated by parasympathetic efferent neurons, as they were abolished in the presence of the muscarinic antagonist atropine. By contrast, the tachycardia was blocked by β-adrenergic antagonists (e.g., metoprolol and propranolol) in the presence of atropine. Moreover, a subset of intrinsic cardiac neurons was found to be responsive to NE in vitro through α-adrenergic receptors [108,109]. These data support a physiological relevance of both parasympathetic and sympathetic efferent neurons in the ICNS [107,108]. Some more specific studies have focused on the action of the intracardiac ganglia on the CCS. Zarzoso and coworkers reported a biphenotypic (adrenergic and cholinergic) action of the pulmonary vein ganglia on the SAN [100]. They employed a right atrial or a Langendorff-perfused heart preparation from mice, together with a combination of adrenergic and cholinergic antagonists (propanol and atropine, respectively). The high frequency stimulation of the pulmonary vein ganglia evoked first a slowing of the heartbeat followed by an acceleration [100]. This biphasic response was explained by various factors: a delayed sympathetic response, the modulation of sympathetic neurons by parasympathetic neurons, and the faster breakdown of acetylcholine [100]. Employing a right atrial preparation and optical mapping, they further demonstrated that stimulation of the pulmonary vein ganglia evoked a downward shift of the leading pacemaker of the SAN in the majority of cases (68.2%) and an upward shift in the minority of cases (31.8%) [100]. These data are in accordance with a dominant parasympathetic response, consistent with other studies that reported either an inferior or superior shift of the leading pacemaker site, in response to the direct stimulation of cholinergic neurons or ACh [110,111,112]. Sampaio and coworkers investigated the effects of the stimulation of two clusters of intracardiac ganglia on the heart rate and atrioventricular conduction in rats. They reported that stimulating the SAN ganglion (the one located between the right atrium and superior vena cava) elicited a bradycardia, but had no effects on conduction; in contrast, the stimulation of the AVN ganglion (located between the pulmonary vein and the right atrium) did not affect heart rate, but slowed conduction [113]. Using subthreshold stimulation in the inferior node extension and compact atrioventricular area, Hucker and coworkers [114] showed that the effects on the AVN resulted from an involvement of both the parasympathetic and sympathetic branches, in contrast to other studies reporting that subthreshold stimulation elicited a purely vagal effect [115]. In contrast to the shifts in the leading pacemaker site in the SAN upon ganglia stimulation, in the rabbit atrial preparation, the location of the AVN pacemaker was found to be stable upon autonomic stimulation [114]. By contrast, a similar protocol resulted in a shift of the AVN pacemaker site in atrial preparations from failing human hearts, i.e., closer to the bundle of His [116].

Overall, these data on the modulation of the ICNS of the SAN and AVN by the ICNS are in accordance with the anatomical distribution of the cardiac ganglia. The dual action of the ganglia innervating the SAN and AVN is consistent with the observation of the presence of both adrenergic and cholinergic neurons in the same ganglia, as discussed in Section 3.2.

### 4.2. Interaction with Peripheral Nerves

The control of the heart by the CANS is hierarchically organized with high levels of interactions within and across the different levels, as mentioned in the introduction and summarized in Figure 1. Notably, using intracellular recordings (in vitro and in vivo) and microelectrode arrays, it was shown that around 40% of neurons in the ICNS receive pre-ganglionic inputs and are classified as principal cells or SAH cells (Figure 4) [85,89,117]. The synaptic transmission from the pre-ganglionic neurons to the post-ganglionic neurons (in the ICNS) has been classified based on the response of excitatory postsynaptic potentials (EPSPs) into three groups: weak (subthreshold), secure, and strong (generally suprathreshold) [90]. In vitro (using the right atrial preparation in conjunction with the vagus nerve), most of the synapses were classified as secure and strong across different developmental stages (neonatal, juvenile, and adult), with only a small proportion being weak [90]. By contrast, using the working heart-brainstem preparation, McAllen and co-workers reported that the synaptic transmission between the pre-ganglionic and post-ganglionic neurons is less than 1:1. A limited number of synapses were, in fact, classified as secure and the majority being strong or weak, some were silent synapses [89]. Notably, the cardiac neurons presenting spontaneous activity were excited by the cardiorespiratory stimuli that elicited bradycardic reflexes (e.g., peripheral chemoreceptors, arterial baroreceptors, and nasotrigeminal receptors). These results were consistent with their role as principal neurons and suggested a convergence of these reflexes on the cardiac ganglia [89]. The contrasting results across the two different preparations might be due to an ongoing synaptic activity in the working heart-brainstem that could have caused a frequency-dependent depression of transmission [89].

As shown in Figure 1, the interactions between the different levels of the CANS are not only between pre- and post-ganglionic neurons, but also through the LCNs. LCNs receive inputs from CNS neurons (e.g., from spinal cord or medulla oblongata), from sympathetic neurons, as well as local afferent neurons. They participate in the transduction of signals within the intrathoracic (intra- and extracardiac) ganglia; this constant communication also persists when chronically disconnected from the central nervous system [2]. LCNs are classified in three main subsets, depending on the cells from which they receive the inputs. The efferent-related LCNs receive inputs from the parasympathetic and sympathetic efferent neurons; the afferent-related transduce regional inputs from local afferent neurons. The third group consists of the convergent LCNs, which integrate information from central efferent (sympathetic and parasympathetic) projections as well as afferent inputs [2,117]. LCNs have also be found to play a role in the initiation and maintenance of tachyarrhythmias. For example, experiments in dogs have shown that the stimulation of mediastinal projections within the ICNS results in atrial fibrillations that can be reduced by selective inhibition of the LCNs [2,118]. In accordance, targeting these subsets of neurons has been suggested as a putative therapy for reducing the nervous system imbalance in cardiac arrhythmias [2].

Cardiac innervation from the stellate ganglia and the vagus nerve presents a high degree of anatomical and functional lateralization. Their lateralization is paired with the GP location in the right and left side of the heart, suggesting a contribution of the intracardiac ganglia in this context [89,119,120]. The right branches of the sympathetic and vagal nerves regulate the SAN, whilst the left branch mainly acts through the AVN influencing ventricular contractility and electrical conduction mainly on the left side [119,120,121]. The role of the GP as integration centers between the extrinsic and intrinsic cardiac nervous system was studied by various groups employing either selective ablation of the GP or via the selective inhibition of nicotinic receptors in the ganglia while using hexamethonium [122,123]. Consistent with the lateralization of the vagal and sympathetic branches, the SAN is controlled by the GP located in the right pulmonary vein, the right atrium, and the superior vena cava, whilst the AVN is regulated by the ones located between the inferior vena cava and the inferior left atrium [122,124,125]. The interactions between the sympathetic and parasympathetic branches are complex. It was reported that reduction in heart rate by vagal stimulation was greater under sympathetic tonic activation [126]. The term “accentuated antagonism” was later coined by Levy [127] to explain the enhanced parasympathetic effect under background sympathetic tone. This is not only observed in case of the heart rate, but also affect the regulation of ventricular performance, intracellular Ca^2+^ levels and cardiac electrophysiology [126]. Various factors might contribute to this response. Muscarinic acetylcholine receptors at the sympathetic terminals inhibit NE release, but adrenergic receptors are not present on parasympathetic terminals [128]. Only under tonic sympathetic activation, the activation of postjunctional muscarinic M2 receptors by parasympathetic terminals results in the inhibition of cAMP production together with the upregulation of PDE2 activity, resulting in cAMP hydrolysis [129]. Apart from the effects at the neurocardiac junction, the accentuated antagonism by the parasympathetic branch can also be modulated at higher brain centers (e.g., cholinergic neurons in the medulla oblongata) [130].

### 4.3. Interaction within the ICNS

It was previously believed that individual GPs were purely responsible for the modulation of the neighboring regions, for example, the right atrial GP for the SAN [7]. However, there is now substantial evidence that the GP can innervate both neighboring regions but also form an intricate network [106,131,132]. Anatomical studies injecting fluorescent tracers in selected ganglia have demonstrated that multiple ganglia can innervate the same cardiac regions and that can also form an inter-ganglionic neuronal circuit [123].

It has been proposed that the short-latency (20–40 ms) reflexes are the results of the action of the neuronal somata in the ICNS located adjacent to cardiomyocytes and, thereby, regulating each cardiac cycle. By contrast longer latency reflexes (100–200 ms) are suggested to be dependent on polysynaptic transmission that involves LCNs modulating efferent neurons for the following cycles also upon stimulus removal [1,3]. Employing decentralized canine hearts, it has been demonstrated that ICNS neurons can work independently from the higher levels. The intrinsic neurons from atrial and ventricular GPs are able to transduce chemical and mechanical modifications: sensing mechanical stimuli from discrete cardiac regions and their spontaneous activity is correlated with the respiratory cycle. Moreover, their spontaneous activity is not modified when the extracardiac tissue is disconnected [133].

Hou and colleagues provided functional evidence for the role of the interplay between various GPs in the regulation of SAN and AVN function [131]. The ablation of selective GPs further demonstrated the collective role of the right atrial and posterior atrial plexi in heart rate regulation. The ones in the right atria directly regulate the heart, whilst the ones on the posterior side are involved in the interaction of the sympathetic and parasympathetic modulation of the heart rate [132].

## 5. Neurocardiac Communication

The foundations of the neuro-cardiac communication have been laid down in the second half of the 20th century employing ultrastructural examinations of heart sections and analyzing the relationship between axon varicosities and the cardiomyocytes [134]. The autonomic neuroeffector communication has long been described as a non-specialized junction that is characterized by a lack of any specific pre- and postjunctional membrane domains with a release of the neurotransmitter *en passage* upon neuronal stimulation [135,136]. However, various studies, especially on sympathetic neurotransmission, suggested a quasi-synaptic mode that is similar, at least to some extent, to the neuromuscular junction (NMJ) [68,137]. The measurement of the distance between the pre- and post-junctional membrane in various species (e.g., toad, guinea pig, mole, and mouse) resulted in the classification of the neurocardiac junction into three groups: intimate (<20 nm), close (20–100 nm), and separated (>100 nm) [67,92,138,139]. Many of these studies were undertaken using random sections; however, when the same varicosity was analyzed in serial sections, most of the varicosities that lost their Schwann cell coating revealed close or intimate contacts [67]. Analyzing serial sections of post-ganglionic varicosities, in the SAN region in amphibian and mammalian species, revealed that the majority of them formed specialized neurocardiac junction with a gap of less than 100 nm in both sympathetic and parasympathetic junctions [67,92]. These junctions presented a high density of synaptic vesicles in the varicosities facing the effector cell and a lower population of vesicles in other parts, with the latter containing larger peptide vesicles, although no pre- or post-synaptic membrane specialization (e.g., thickening) was observed [67,92] (Figure 4).

### 5.1. The Sympathetic-Cardiac Junction

Sympathetic neurons release NE and, together with epinephrine released by the adrenal medulla, act on adrenergic receptors in the heart. β1-, β2-, and β3- adrenergic receptors (ARs) are expressed in the heart, with higher overall levels of β1ARs and they are all G protein-coupled receptors (GPCRs) [140]. The βARs signaling cascade downstream of the βARs is distinct: β1AR couples to G_s_, eliciting an increase in cAMP and inducing an activation of its downstream effector proteins. By contrast, β2 and β3ARs can act on both G_s_ and G_i_, with the latter being involved in the attenuation of the cAMP signaling with β2ARs able to modulate β1AR signaling [140]. The activation of β3ARs can also result into cGMP production via eNOS activation [140,141]. Another factor influencing their downstream effects is the microdomain localization with β1ARs being widely distributed in the sarcolemma and β2- and β3ARs mostly confined to T-tubules in ventricular myocytes, with receptor redistribution occurring in disease, e.g., in heart failure [141,142]. Cardiomyocytes also express α1-adrenoceptors (α1a and α1b), which are coupled to G_q/11_. Their activation results in positive inotropic effects, and chronic stimulation induces hypertrophy [140].

As mentioned in Section 3.2, a subset of intrinsic cardiac neurons has been found to express the enzymes that are involved in catecholamine biosynthesis [99]. Moreover, as mentioned in Section 4.1 a subgroup of intrinsic neurons has been reported to elicit a tachycardia effect, which was blocked by βARs antagonists further supporting their functional relevance [2,108]. The sympathetic neuro-cardiac communication has been mainly investigated in vitro using, as a model system, co-cultures of neonatal cardiomyocytes with sympathetic neurons that are isolated from superior cervical or stellate ganglia. It has been reported that sympathetic neurons in vitro can differentiate into cholinergic or adrenergic neurons [143]. Neurotrophic factors can alter their fate (e.g., BDNF induces cholinergic differentiation [144]) and that NGFs strengthen the excitatory transmission between sympathetic neuron and cardiomyocytes in culture [45,46]. Many groups that use NGF in the culture medium reported nearly exclusive catecholamine release from sympathetic neurons, recapitulating in vitro sympathetic innervation, and revealed the structure and function of the sympathetic neurocardiac junction [45,46,145,146]. In axons, which are in contact with cardiomyocytes, activity-dependent recycling of synaptic vesicles can be observed, together with the presence of markers for neuroexocytosis in the prejunctional region (e.g., synapsin 1, synaptosomal-associated protein 25 (SNAP25), and synaptotagmin) [145,146]. The post-junctional cardiomyocyte membrane is characterized by the presence of scaffolding proteins (e.g., synapse-associated protein 97 (SAP97), A kinase anchoring protein 79 (AKAP79), and ankyrin G) and molecular complexes potentially stabilizing cell-cell adhesion (e.g., cadherin and β-catenin) both in vitro and in vivo [145,146,147]. Moreover, the postjunctional membrane reorganization was also defined by the enrichment of specific channels (e.g., voltage gated sodium channel 1.5 (Na_v_1.5) and the voltage activated K^+^ channel subfamily Q member 1 (KCNQ1)) and receptors (e.g., β1ARs), with the exclusion of caveolin-3 at contact sites and the removal of β2ARs upon neuronal stimulation [145,147]. Interestingly, in accordance with a role of β1ARs in the post-junctional membrane, the tachycardia that is induced in pacemaker cells by sympathetic neural stimulation is mostly blocked by a β1AR-selective antagonist. In contrast, little inhibition is elicited by β2ARs antagonists in a guinea pig atrial preparation in contiguity with the thoracic ganglia [148]. Evidence of distinct functional pools of adrenergic receptors at extra-junctional and post-junctional sites was also obtained using the toad atrial preparation and the catecholamine reuptake inhibitor desmethylimipramine (DMI) to allow for the escape of catecholamines from the junction [149].

In sympathetic neuron-cardiomyocytes co-cultures, NE release in the neurocardiac junction peaks at around 200 ms after neuronal stimulation and declines after 460 ms. The time-course of NE in the cleft is mainly determined by the NE reuptake transporter (NET) in the prejunctional membrane [150]. NET dysfunction has been reported in various cardiac diseases. Polymorphisms of the human gene encoding NET-1 (*SLC6A2)*, which are often associated with a reduction of its function, have, for example, been associated with postural tachycardia syndrome and congestive heart failure [151]. In accordance with the release of NE in a “diffusion-restricted” manner, in sympathetic neuron-cardiomyocyte co-cultures, NE concentration was estimated to be around 100 nmol L^−1^ in the cleft resulting in a spatially restricted increase of cAMP in the innervated cardiomyocyte [146]. Furthermore, the prejunctional membrane presents various receptors that are involved in the regulation of NE release, with β2ARs facilitating and α2-adrenergic receptors inhibiting the release [152,153,154]. Notably, in atrial preparations from mammals and amphibians, the adrenergic responses in the pacemaker cells elicited by stellate ganglia stimulation do not appear to be mediated by the cAMP-dependent pathways. However, catecholamines bath application, in the same preparation, elicited an increase in cAMP [148,149]. Interestingly, studies employing Förster resonance energy transfer (FRET) sensors in co-cultures of sympathetic neurons and cardiomyocytes revealed an increase of cAMP in cardiomyocytes, which were in direct contact with the stimulated neuron [146]. The rise in cAMP levels was faster and greater in the region of the cardiomyocyte proximal to the neuronal contact site when compared to distal regions [146]. These findings are consistent with the highly compartmentalized nature of cAMP signaling, which is characterized by changes in nanodomain signaling upon localized stimulation [142]. This could perhaps explain the absence of a cAMP response at the whole-cell level when pacemaker cells were recorded upon stellate ganglia stimulation. Further experiments are needed and employing for example FRET sensors targeted to distinct cellular microdomains [155]. However, a putative involvement of cAMP-independent pathways downstream of β-adrenergic stimulation should not be excluded. Accordingly, in rabbit cardiomyocytes, a direct effect of isoproterenol causes an enhancement of the Na^+^ current (I_Na_) via a G_s_-coupled pathway, which is independent of cAMP signaling [156].

Apart from the effects on pacemaker cells, sympathetic neurons also modulate the contractility of rodent cardiomyocytes [46,157,158] and induced pluripotent stem cells (iPSC)-derived cardiomyocytes [159,160]. Sympathetic innervation in the murine heart is more abundant in the epicardial layer and it diminishes towards the endocardium [161,162,163]. This transmural innervation gradient may be responsible for size difference of cardiomyocytes in the epicardial and endocardial layer. Accordingly, a trophic role of sympathetic neurons on cardiomyocytes has been reported [164]. Sympathetic innervation also plays a role in cardiac regeneration in neonatal mouse with sympathetic cardiac denervation completely inhibiting this process [165].

### 5.2. The Parasympathetic-Neurocardiac Junction

In the heart, acetylcholine that is released by nerve terminals acts on the metabotropic muscarinic acetylcholine receptors, which are classified into M1–5. M2 is highly expressed in cardiac tissue and is coupled to G_i_, resulting into a decrease in cAMP signaling. There is also some evidence of the presence of M1 and M3 in the heart, which, in contrast to M2, are coupled to G_q/11_ and trigger the PLC signaling pathways [140]. Evidence for the formation of functional neurocardiac junctions between parasympathetic neurons and myocytes was obtained in neuron-myocytes co-cultures. Stimulation of the parasympathetic neurons (isolated from sacral cord explants or ciliary muscle or iPSC-derived) elicits a negative chronotropic effect in innervated cardiomyocytes (ventricular, sinoatrial node, or iPSC-derived) [160,166,167,168]. In neurocardiac co-cultures from either rat or chicken, the functional neuromodulation of cardiomyocytes develops after three days in culture. The mere vicinity between neurons and cardiomyocytes does not necessarily implicate the formation of functional contacts [167,168]. An increase in the expression levels of specific G_α_ subunits is concomitant with the enhancement of the muscarinic responsiveness of the cardiomyocytes only when co-cultured with neurons [168]. Similar to the sympathetic neurocardiac junction, changes in protein localization in the pre- and postjunctional membrane may occur upon parasympathetic neurocardiac junction formation and neuronal stimulation or triggered by molecules that are involved in cell–cell adhesion. Although this has not been investigated in the heart, various cadherins have been reported to be involved in parasympathetic neuroeffector junction formation. Accordingly, the expression of *N*-cadherin in CHO cells induces a partial differentiation of presynaptic cholinergic terminals of brainstem neurons when cell–cell contact are established [169].

The parasympathetic neurocardiac junction at the level of the SAN has been studied by the Hirst group in an atrial preparation in contiguity with the vagus nerve using intracellular recordings of the sinoatrial pacemaker cells, in order to compare the effects of either ACh application or vagal stimulation [85,170,171]. Both treatments result in a decrease in action potential generation and beating rate. ACh also elicits membrane hyperpolarization, action potential shortening, and an increase in its peak potential, whilst vagus nerve stimulation induces membrane depolarization and has little or no effects on the peak potential and its duration [170,171,172]. Using pharmacological tools, e.g., cesium for blocking the funny current channels and Ba^2+^ for K^+^ current channels, they proposed the existence of post-junctional and extra-junctional muscarinic receptors, with both effects being inhibited by muscarinic antagonists. Vagal stimulation result in the reduction of the funny current (i*_f_*) without an involvement of the K^+^ channels (i.e., I_KACh_) downstream of a pool of muscarinic postjunctional receptors, whilst bath application of ACh activates also muscarinic extra-junctional receptors that are coupled to K^+^ channels [134,170,171,172]. The distinct effects of exogenous application of agonists compared to neuronal stimulation, debunked the idea that the exogenous application of agonist mimics physiological conditions [4]. Other groups assessing the beating rate of a mammalian atrial preparation using Ba^2+^ [173] or the diastolic tension in the preparation while using the selective I_KACh_ blocker tertiapin-Q [174] obtained different results. They described an involvement of the I_KACh_ downstream of the muscarinic receptor upon vagal stimulation. A contribution of I_KACh_ was also postulated by the reduction of cardiac autonomic regulation in a mouse mutant lacking Kir3.4 channels [175]. These results raise questions regarding the contribution of IK_ACh_ in pacemaker cells upon vagal stimulation. Do these channels reside at the post-junctional membrane or are they activated by, for example, spillover ACh? The carefully executed experiments by the Hirst group should not be refuted without reproducing them [176]. When comparing the experimental results across different studies, it is also important to keep in mind the structural heterogeneity of SAN tissue with distinct densities of ion channels, innervation levels, and the expression of different connexin isoforms [177].

At the level of the parasympathetic neurocardiac junction, acetylcholine in the cleft is cleaved by AChE, which is a hydrolytic enzyme that is present at both pre- and postsynaptic membranes. Choline, which results from this enzymatic reaction, is readily transported back into the neuron by a high affinity choline uptake mechanism, ACh can then be re-synthesized by choline acetyltransferase and subsequently released again [135]. The AChE levels are higher surrounding the SAN when compared to atrial muscle and they are even lower in the ventricles [178]. The lower AChE levels in atrial tissue are probably the reason for the slower rate of recovery in atrial muscle after vagal nerve stimulation [179]. There is some evidence using the right atrial preparation that acetylcholine can inhibit its own release from parasympathetic terminals through its action on muscarinic autoreceptors. In accordance with this, the muscarinic receptor antagonist atropine strongly increases ACh release from the vagus nerve in the right atrial preparation [180].

Apart from the role of cholinergic neurons in action potential modulation, they have also been implicated in cardiac regeneration in both zebrafish and neonatal mice. Their pharmacological or genetic blockade prevents cardiac regeneration upon nerve injury, resulting in a decrease of cardiomyocyte proliferation paired with a decrease of cell cycle genes [181].

### 5.3. Co-Transmission

**Figure 5:**
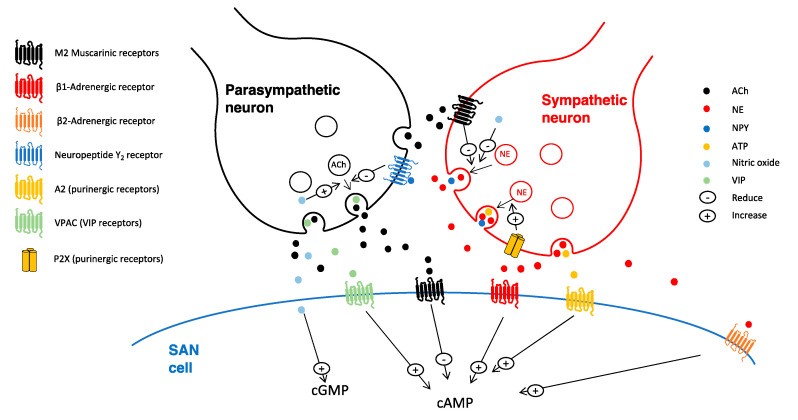
Diagram illustrating the crosstalk by the sympathetic and parasympathetic varicosities at the level of a sinoatrial node myocyte. The parasympathetic neuron can release acetylcholine, nitric oxide and vasoactive intestinal peptide, whilst the sympathetic neuron norepinephrine, ATP and neuropeptide Y. This figure was created using images from Servier Medical Art Commons Attribution 3.0 Unported License (http://smart.servier.com). Servier Medical Art by Servier is licensed under a Creative Commons Attribution 3.0 Unported License.

The idea according to which neurons could only release one neurotransmitter, known as “Dale’s principle”, was first challenged by Geoffrey Burnstock in 1976, who suggested that neurons of the same class could release more than one neurotransmitter [182]. Dales’ principle was hence rectified by Sir John Eccles into ‘at all the axonal branches of a neuron, there is liberation of the same transmitter substance or substances’ [183]. Sympathetic neurons, in addition to NE, can release neuropeptide Y (NPY) and adenosine triphosphate (ATP), with distinct stimulation parameters favoring some neurotransmitters over others: e.g., low frequency favors ATP release, longer period NE, and high frequency with sporadic burst towards NPY [135]. In contrast, parasympathetic neurons can release, in addition to ACh, vasoactive intestinal peptide (VIP), ATP, and NO [135]. Figure 5 summarizes the co-transmitters released at the neurocardiac junction and the cross-talks between the sympathetic and parasympathetic varicosities at the level of the sinoatrial node.

NPY can be released together with NE and it can inhibit cholinergic release [183]. The analyses of the perfusate from the atrial preparation in vitro and the coronary sinus blood in vivo demonstrated that electrical stimulation protocols of sympathetic neurons (e.g., stellate ganglia). known to inhibit the vagal bradycardia, result in the release of NPY [183,184]. NPY acts on GPCRs that are coupled to the G_i/o_ pathway. Y_1_, Y_2_ and Y_5_ receptors are expressed in the heart and neuropeptide Y detected in the CCS, atria and coronary vessels [185]. NPY receptors have been reported to be present both at presynaptic sites inhibiting neurotransmitter release as well as postjunctional sites enhancing the NE effects [185]. In the heart, the full action of NPY is not clear, with inconsistent changes in heart rates [186]. Y_1_ receptors are found in ventricular cardiomyocyte and they enhance the response of NE [183,184]. In contrast, Y_2_ receptors are expressed in prejunctional cholinergic terminals (e.g., from intrinsic cardiac neurons as shown in the guinea pig atrial preparation) and upon activation they inhibit ACh release via a PKC-dependent pathway [104,183,184]. Y_2_ receptors in these terminals have been shown to be functionally active, as NPY application in the atrial preparation in conjunction with the right vagal nerve reduced the heart rate response to vagal stimulation and decreased acetylcholine release [104,184]. Moreover, these effects were blocked by Y_2_ antagonists or through genetic ablation [183,184].

Some intrinsic cardiac somata and fibers in the guinea pig atria are found to be reactive to quinacrine, which is a marker of high ATP content in vesicles containing neuropeptides. These findings suggested that these neurons could release ATP [187]. Purinergic receptors binding ATP are classified into P1 (A_1_, A_2A_, A_2B_ and A_3_) and P2 receptors. P2 receptors are sub-categorized into P2X (P2X_1–7_) and P2Y (P2Y_1,2,4,6,11,12,13,14_). P1 is most sensitive to adenosine, which results from ATP breakdown, whilst P2 to ATP. P2X receptors are inotropic (ligand-gated ion channel) receptors, whereas P1 and P2Y GPCRs [135]. A_1_ and A_3_ receptors are coupled to G_i_ and G_q/11_ pathways mimicking downstream effects that are similar to muscarinic M_2_ receptors, whilst A2 receptors are coupled to Gs; the sinoatrial node expresses A1 receptors [186]. Adenosine and ATP reduce the pacemaker activity of the SAN, the conduction of the AVN, and the automaticity of the His bundle and Purkinje fibers. In human patients, intravenous administration of ATP produces AV block via P1 receptors. In patients with paroxysmal supraventricular tachycardia, bolus injection of adenosine (Adenocard) is employed in order to reduce the conduction time of the AVN [187]. In the heart, the P2 receptor has a distinct spatial distribution with P2X1/3/4/5/6 being expressed in the ventricles, whilst P2X1-6 in the atria [188]. P2X activation and the resulting increase of intracellular cations (e.g., Na^+^) elicits cardiomyocyte contractility [189]. P2X receptors are also found in the prejunctional regions of cardiac sympathetic neurons, and their activation promotes NE release [190]. Studies of sympathetic neuro-effector junctions of smooth muscle cells have reported that, when ATP is co-released with NE, the action of ATP on the effector cell is faster due to the faster P2X receptor response as compared to the slower effects of NE via GPCRs [146,150].

A subset of intracardiac neurons present P2Y_2_ receptors and they are sensitive to ATP application and, to a lesser extent, to ADP (but not to AMP or adenosine) [187], although there is some evidence of a putative role of P1 receptors in prejunctional regions [191].

The application of ATP elicits distinct effects on intrinsic cardiac neurons via P2Y receptors, resulting in three different responses. In the first group of cells, it elicits a rapid transient depolarization; in the second group, the depolarization is followed by an hyperpolarization and a slow prolonged depolarization; in the third subset, ATP induces a slow depolarization [187,192]. These results suggest that some intrinsic cardiac neurons are local afferent neurons, playing a role in the regional cardiac reflexes, in accordance with this, nodose afferent neurons can be activated by ATP and adenosine [187,192].

VIP-positive fibers are present in high density around the SAN of all mammals [193]. Despite being released from parasympathetic terminals, VIP has the opposite effects of ACh i.e., it increases the synthesis of cAMP, which results in the enhancement of the heart rate. It has been suggested to be involved in postvagal tachycardia, as its removal from the neuroeffector junction is slower when compared to ACh [175]. Vagal nerve stimulation in the presence of muscarinic and adrenergic antagonist results in an increase in heart rate. This effect was found to be mediated by VIP receptors, as their antagonists prevented it and it was mimicked by VIP application [193]. VIP acts on two receptor types (VPAC1 and VPAC2), both being expressed in the heart; they are both G_s_-coupled GPCRs. In SAN, AVN, and Purkinje fibers, VIP application results in a positive shift in the funny current activation curve, resulting in a chronotropic effect [175,186]. VIP receptor distribution is affected in various diseases (e.g., heart failure and hypertension) also affecting their responsiveness to agonists [186].

Nitric oxide (NO) is a fundamental intra/intercellular signaling molecule that can act in an autocrine/paracrine manner [101,194]. NO is tonically released and it can reach 1–3 μM during diastole. NO has a dual action on its modulation of heart rate. Its action on prejunctional receptors facilitates ACh release and decreasing heart rate, whilst its postjunctional action on I*_f_* can increase heart rate [101]. NO can modulate neurotransmitter release from autonomic neurons: it inhibits NE and facilitates ACh release [101]. It can facilitate the cholinergic suppression of βARs signaling in cardiomyocytes, although there is some debate on its physiological action [194]. It is produced from L-arginine by nitric oxide synthases. There are three isoforms of this enzyme with distinct cellular expression: eNOS (in endothelium and plasma membrane of cardiomyocytes), nNOS (neurons and sarcoplasmic reticulum of cardiomyocytes), and iNOS expressed during inflammation [140]. NO increases the intracellular cGMP production by binding to the soluble form of guanylyl cyclase, which, in turn, activates various signaling partners e.g., PKG and PDEs modulating heart rate, for example, by reducing I_CaL_ in the SAN [186]. There is some controversy regarding the functional effects of nNOS on heart rate regulation of parasympathetic neurons [101]. It has been reported that nNOS levels vary across development with an increase of nNOS in older animals, which might explain the discrepancy of the data in the literature [101]. In accordance to changes across developmental stages, Herring and coworkers have shown that the inhibition of nNOS and guanylyl cyclase significantly reduces the heart rate response of vagal stimulation on the atrial preparation in older guinea pigs, but not in younger ones [195]. It seems that the effects of NO on heart rate, are mainly through its action on prejunctional receptors. Sodium nitroprusside (the nitric oxide donor) increases ^3^H-ACh release, the application of NO activators in the presence of carbamylcholine (the stable analogue of ACh), and NOS and guanylyl cyclase inhibitors do not result in any changes in heart rate [101].

## 6. Cardiac Innervation and Heart Disease

Disruption in cardiac innervation has been implicated in various disorders (as summarized in Table 1), for example, dysautonomia (i.e., diseases arising from disrupted autonomic interactions), as well as in cardiac arrhythmia (e.g., in atrial fibrillation). In other cases, for example, after heart transplantation, the cardiac afferent and efferent neurons (to and from the extrinsic cardiac nervous system) are completely removed. This results in the loss of the functional interaction with the stellate ganglia and vagus nerve and, ultimately, causes a depletion of the modulation by the higher brain centers.

Anatomical studies of the human heart have shown age-dependent changes of innervation. It has been reported that, during infancy, there is a dominance of sympathetic innervation of the CCS. There is a codominance of sympathetic and parasympathetic innervation during adulthood and an overall reduction of innervation in the elderly, with a specific reduction of parasympathetic function [196]. Moreover, anatomical and functional studies using aged mice reported a reduction in sympathetic heart rate regulation in the atria and ventricles of aged animals, which was the result of a reduction in ventricular sympathetic innervation and atrial β-adrenergic responsiveness [197].

The most common forms of dysautonomia are the familial ones. Patients with familial dysautonomia express low levels of dopamine-beta-hydroxylase resulting in a reduction of NE synthesis. This syndrome has been associated with mutations in the inhibitor of kappa light polypeptide gene enhancer in B-cells, kinase complex-associated protein (*IKBKAP)* gene. Individuals with familial dysautonomia have an abnormal heart rate, cardiac tone, and blood pressure together with higher risk of sudden death [198,206,207]. Another disease that is associated with abnormal development of the autonomic nervous system, possibly due to delayed maturation, is sudden infant death syndrome (SIDS), which is characterized by a reduction of the cardiac sympathovagal balance and anomalies in sleeping pattern [198].

Atrial fibrillation arises from multiple factors, among them: elevated vagal tone and ectopic firing in the region that surrounds the pulmonary vein. This region contains various intrinsic cardiac ganglia and myocytes with specific electrophysiological properties making them more susceptible to arrhythmogenesis [200,208]. Moreover, the stimulation of GP in the left atrial fat pad lowers the threshold for atrial fibrillation, whilst their ablation can prevent it [199,200]. However, because of the intricated GP network, their role in physiological regulation of the CCS, innervation, and modulation of ventricles, ablation procedures can have serious side-effects, among them ventricular fibrillation [209]. Furthermore, neuronal remodeling (e.g., an increased presence of sympathetic neurons) has been reported in atrial fibrillation, which could have an effect on the outcome of ablation therapy [7].

Some studies investigating the pathophysiology of sick sinus syndrome in patients reported that the disease phenotype was mainly the result of an intrinsic abnormality of the SAN. Nonetheless, in some cases it was secondary to increased autonomic activity, in fact, autonomic blockade restored normal heart rate [202,203]. From the patient pathological phenotypes, it was difficult to distinguish whether the SAN bradycardia was the result of SAN or autonomic nervous system dysfunction [202]. It is also important to note that, some ion channels, which have been associated with cardiac arrhythmogenic disorders, are expressed not only in cardiomyocytes, but also in intracardiac ganglia. One example is the sodium channel Na_v_1.5, whose functional expression has been reported in canine intracardiac ganglia implying a potential role of the ICNS in the pathophysiology of various disorders [201]. In accordance with this, mutations in its gene (*SCN5A*) have been associated with various cardiac arrhythmic disorders, e.g., long QT syndrome 3 (LQT3), Brugada syndrome and progressive conduction disease [201]. LQT3 is often linked to SAN dysfunction (e.g., sinus bradycardia and pauses), together with atrial standstill [201]. A number of studies on the dysfunction of the CCS have focused on ion channels, transmembrane and membrane-associated proteins in the SAN e.g., ankyrin B [111], Ca_V_1.3, Ca_V_3.1 [210], GIRK [211], POPDC1 [212], POPDC2 [213], or studied the effects of muscarinic or βAR agonists and/or antagonists on SAN excitability [111,210]. However, little work has been undertaken so far on the putative contribution of the ICNS in these diseases. There are some preliminary data on a potential role of the cAMP-binding transmembrane protein POPDC1 in the ICNS (unpublished data). Mutations in the *POPDC1* gene have been associated with skeletal muscle and cardiac disorders, specifically causing a disruption of CCS function e.g., atrioventricular block in patients and zebrafish mutants and a stress-induced sinus bradycardia in mutant mice [212,214]. Interestingly, *popdc1* expression has recently been detected in the ICNS of the zebrafish (unpublished data). The recessive *POPDC1^S201F^* missense mutation causes a second-degree atrio-ventricular block in patients [214]. A subset of zebrafish mutants carrying the homologous mutation (*popdc1^S191F^*) showed electrocardiogram (ECG) changes at baseline (atrioventricular block) [214]; isoproterenol and neuronal stimulation elicited arrhythmia, including sinus pauses (unpublished data). Notably, fishes with the most severe abnormalities also presented dysfunctions at the level of the ICNS. Significantly, the phenotype was rescued by cholinergic agonist suggesting a role of POPDC1 for the proper ICNS function (unpublished data). Interestingly, Mangoni and coworkers undertook extensive research on mouse models with SAN and AVN dysfunction and reported a rescue of the pathological phenotype after either pharmacological blockade or genetic ablation of the IK_ACh_ channel (GIRK4), which acts downstream of the muscarinic receptors [210,215]. These data suggest distinct pathophysiological mechanisms that are involved in conduction disorders with a beneficial role of either muscarinic agonist or antagonist, depending on the underlying disease mechanisms. Until now, the question of the role of the ICNS or the cardiac nervous system, in general, in heart rhythm disorders has not been sufficiently experimentally addressed. Further studies involving, for example, the conditional ablation of genes in neurons and SAN myocytes could reveal the potential contribution of the ICNS in these disorders.

Heart transplantation is characterized by the surgical interruption of the extrinsic efferent and afferent neurons to and from the heart (e.g., vagal input, extrinsic cardiac ganglia) leading to axonal degeneration and impairments of the cardiac reflexes [204]. Abnormal afferent-efferent communications have also been reported in various diseases, for example, in ischemic ventricles resulting in an increased susceptibility of sudden cardiac death [2].

Because of the allograft denervation, abnormal cardiac function at rest and during exercise, e.g., higher heart rate, slower changes in heart rate, and abnormal cardiac output, have been reported [204]. After heart transplantation, it has been reported that the intracardiac neurons preserve synaptic neurotransmission, but they undergo changes in membrane properties, e.g., reducing their afterhyperpolarization current, hence increasing the proportion of phasic neurons, and possibly increasing their excitability [205]. Interestingly, in 40–70% patients, partial restoration of the innervation after heart transplantation has been reported, but is sometimes defined as partial or patchy reinnervation, with some patients presenting little or no innervation after 10 years [204]. The sympathetic branch can arise after six months and the parasympathetic after 1–3 years following sympathetic innervation [204]. The delay in the parasympathetic reinnervation can result into unbalanced autonomic innervation causing abnormal heart rate and response to stimuli [204]. Sympathetic ventricular innervation was reported to be time-dependent starting from the base towards the apex, whilst innervation of the sinoatrial node was more variable [204]. Another factor affecting adrenergic-cholinergic balance is the upregulation of the neural-crest derived ICA cells, which, however, has only been reported so far in mice [22]. Given the partial innervation after heart transplantation and the resulting abnormal sympathetic-parasympathetic balance, promoting cardiac innervation opens up a research scope for tissue engineering, e.g., triggering neo-innervation before heart transplantation or prior to implantation [216].

## 7. Conclusions

There is increasing appreciation of the important function of the ICNS in the modulation of heart function through its regulation of the CCS. However, the full degree of its physiological action and how it is affected in various cardiovascular disorders remains to be properly studied. There are still many open questions that need to be addressed.

The cardiac parasympathetic innervation as compared to sympathetic innervation is generally understudied. For example, during development, some information has been extrapolated from ciliary ganglia and, therefore, might not be fully correct. Various animal models could be employed in order to address this question; one could, for example, take advantage of the transparency of the zebrafish embryo, as the zebrafish has recently been established as a novel model for studying the ICNS [78].

Despite evidence of a neurocardiac junction similar to the sympathetic branch, less work has been undertaken for the parasympathetic junction. Moreover, the protocols often used to study the effect of sympathetic and parasympathetic innervation involved bath application of agonists/antagonists, which does not recapitulate the physiological condition. It is important to keep in mind the role of co-transmitters and the structure of the neurocardiac junction with a differential distribution of channels and receptors at post-junctional versus extra-junctional membrane domains. The neurocardiac junction could be further studied, for example, employing FRET sensors localized to distinct cellular microdomains in neuron-cardiomyocyte co-cultures. Moreover, co-cultures of iPSC-derived neurons and cardiomyocytes form functional neurocardiac junctions and may also permit modelling various neurocardiac diseases while using patient-derived cells.

Despite the advances in the classification of neurons of intracardiac ganglia based on their molecular profile, their biophysical profile is yet to be paired to their neurochemical characteristics. This could be achieved, for example, using Patch-seq, as already mentioned in Section 3.2. Elucidating the transcriptome of ICNS neurons could reveal whether some genes that are known to be involved in the pathophysiology of cardiovascular diseases (e.g., *SCN5A* in Brugada syndrome) are also expressed in the ICNS. Further insights in their molecular expression profile in combination with the generation of animal models with neuron or cardiomyocyte-specific ablation of genes will allow for the assessment of the contribution of different cell types in the disease pathology possibly resulting in the identification of novel drug targets. Further research is also needed in order to study the role of the ICNS in atrial fibrillation. Tissue engineering approaches may help to restore cardiac innervation after heart transplantation.

Research on the ICNS has long been a neglected area in cardiac biology. However, with the recent development of single-cell sequencing and the availability of marker genes for cell sorting, it is likely that we will soon have deeper insight into the makeup of the ICNS and its role in health and disease.

## Figures and Tables

**Table 1:** Summary of the contribution of the ICNS in some cardiac diseases.

Disease	Involvement of the ICNS
Ageing	General reduction of parasympathetic and sympathetic function in aged animals [196,197]
Dysautonomia	General reduction of NE synthesis. Specific contribution of the ICNS not known [198].
Atrial fibrillation	Stimulation of GPs can elicit whilst their ablation can prevent atrial fibrillation [199,200], evidence of neuronal remodeling [7]
Conduction disorders	Expression of some “cardiac” proteins associated with the disease pathology in the ICNS [201]. Evidence of pathology secondary to increased autonomic activity [202,203].
Heart transplantation	Loss of the extrinsic innervation (efferent and afferent) to/from the heart [204]; changes in ICNS membrane properties [205]

## Abbreviations

β1AR	beta1 adrenergic receptor
β2AR	beta2 adrenergic receptor
ACh	acetylcholine
AChE	acetylcholinesterase
ATP	adenosine triphosphate
AVN	atrioventricular node
BMP	bone morphogenetic protein
CANS	cardiac autonomic nervous system
CCS	cardiac conduction system
CGRP	calcitonin gene related peptide
ChAT	choline acetyltransferase
DBH	dopamine β-hydroxylase
ECG	electrocardiogram
ET-1	endothelin-1
FRET	Förster Resonance Energy Transfer
GATA3	GATA binding protein 3
GDNF	glial cells neurotrophic factors
GFL	Glial cell-line derived neurotrophic factor family of ligands
GP	ganglionated plexi
GPCRs	G-protein coupled receptors
HAND2	heart and neural crest derivative expressed 2
ICA	intrinsic cardiac adrenergic
ICNS	intrinsic cardiac nervous system
IPSCs	induced-pluripotent stem cells
IKBKA	kinase complex-associated protein
LCNs	local circuit neurons
MASH1	mammalian achaete-scute homolog
M2	muscarinic acetylcholine receptors type 2
NCCs	neural crest cells
NE	norepinephrine
NET	norepinephrine reuptake transporter
NGF	nerve growth factor
nNOS	neuronal nitric oxide synthase
NO	nitric oxide
NOS	nitric oxide synthase
NPY	neuropeptide Y
PHOX2A	paired- like homeobox 2a
PHOX2B	paired- like homeobox 2b
PNMT	phenylethanolamine-*N*-methyltransferase
SAN	sinoatrial node
SAR	sinoatrial ring
SIF	small intensely fluorescent cells
SP	substance P
TH	tyrosine hydroxylase
vAChT	vesicular acetylcholine transporter
VMAT-2	vesicular monoamine transporter 2
VIP	vasoactive intestinal peptide
